# Indirect Immobilised Jagged-1 Enhances Matrisome Proteins Associated with Osteogenic Differentiation of Human Dental Pulp Stem Cells: A Proteomic Study

**DOI:** 10.3390/ijms232213897

**Published:** 2022-11-11

**Authors:** Ajjima Chansaenroj, Chatvadee Kornsuthisopon, Sittiruk Roytrakul, Suphalak Phothichailert, Sunisa Rochanavibhata, Benjamin P. J. Fournier, Supreda Suphanantachat Srithanyarat, Nunthawan Nowwarote, Thanaphum Osathanon

**Affiliations:** 1 Dental Stem Cell Biology Research Unit, Faculty of Dentistry, Chulalongkorn University, Bangkok 10330, Thailand; 2 National Center for Genetic Engineering and Biotechnology, National Science and Technology Development Agency, Pathum Thani 12120, Thailand; 3 Department of Oral and Maxillofacial Surgery, Faculty of Dentistry, Chulalongkorn University, Bangkok 10330, Thailand; 4 Centre de Recherche des Cordeliers, Université Paris Cité, Sorbonne Université, INSERM UMR1138, Molecular Oral Pathophysiology, 75006 Paris, France; 5 Department of Oral Biology, Faculty of Dentistry, Université Paris Cité, 75006 Paris, France; 6 Department of Periodontology, Faculty of Dentistry, Chulalongkorn University, Bangkok 10330, Thailand; 7 Department of Anatomy, Faculty of Dentistry, Chulalongkorn University, Bangkok 10330, Thailand

**Keywords:** extracellular matrix, human dental pulp stem cells, indirect immobilisation Jagged-1, proteomics

## Abstract

The indirect immobilisation of Jagged-1 (Jagged-1) promoted osteogenic differentiation of human dental pulp cells (hDPs). Furthermore, the analysis of the Reactome pathway of RNA sequencing data indicates the upregulated genes involved with the extracellular matrix (ECM). Hence, our objective was to investigate the effects of Jagged-1 on proteomic profiles of human dental pulp stem cells (hDPSC). hDPSCs were cultured on the surface coated with human IgG Fc fragment (hFc) and the surface coated with rhJagged1/Fc recombinant protein-coated surface. Cells were differentiated to the osteogenic lineage using an osteogenic differentiation medium (OM) for 14 days, and cells cultured in a growth medium were used as a control. The protein component of the cultured cells was extracted into the cytosol, membrane, nucleus, and cytoskeletal compartment. Subsequently, the proteomic analysis was performed using liquid chromatography–tandem mass spectrometry (LC-MS). Metascape gene list analysis reported that Jagged-1 stimulated the expression of the membrane trafficking protein (DOP1B), which can indirectly improve osteogenic differentiation. hDPSCs cultured on Jagged-1 surface under OM condition expressed COL27A1, MXRA5, COL7A1, and MMP16, which played an important role in osteogenic differentiation. Furthermore, common matrisome proteins of all cellular components were related to osteogenesis/osteogenic differentiation. Additionally, the gene ontology categorised by the biological process of cytosol, membrane, and cytoskeleton compartments was associated with the biomineralisation process. The gene ontology of different culture conditions in each cellular component showed several unique gene ontologies. Remarkably, the Jagged-1_OM culture condition showed the biological process related to odontogenesis in the membrane compartment. In conclusion, the Jagged-1 induces osteogenic differentiation could, mainly through the regulation of protein in the membrane compartment.

## 1. Introduction

Human dental pulp stem cells (hDPSCs) are derived from ectodermal neural crest cell origin and possess mesenchymal stem cell traits [1]. hDPSCs have been extensively studied for their application in tissue engineering and regenerative medicine [2,3,4,5,6,7] owing to their non-invasive accessibility and multilineage differentiation ability [8,9,10]. In the bone regeneration field, hDPSCs exhibit superior mineralisation potency over adipose tissue and bone marrow cells [11]. Hence, there are numerous studies on the mechanisms/pathways involved in hDPSC differentiated into the osteogenic lineage.

Notch signalling is one of the crucial pathways that influence osteogenic differentiation. In general, Notch signalling plays a pivotal role by governing stem cell proliferation and differentiation and participates in many cellular processes, including skeletal tissue development, tissue homeostasis, regeneration in various cell types, and also cancer cell regulation [12,13,14,15,16,17,18]. Transmembrane Notch receptors on the cell membrane transduce the signals by interacting with transmembrane Notch ligands on the neighbouring cells via cell-to-cell binding [19]. The binding leads to the cleavage of the Notch receptor at the intracellular site and then to the release of the Notch intracellular domain (NICD). NICD further translocates into the nucleus to regulate the Notch target gene expression [20].

Among Notch ligands, Jagged-1 drives osteogenic differentiation in various oral tissues [19,21,22,23,24]. Immobilised Jagged-1 mediated Notch activation significantly promotes the differentiation markers of epithelial stem cells compared with a soluble Jagged-1 [25]. Similarly, a previous study reports that indirect immobilised Jagged-1 (Jagged-1) is the most effective approach to activation of Notch signalling that significantly activated *HES1* and *HEY1* among direct immobilisation, indirect immobilisation, and soluble Jagged-1 [23]. Moreover, the Jagged-1 promotes mineral deposition of human dental pulp cells (hDPs) and stem cells isolated from human exfoliated deciduous teeth [21,24,24,26,27]. Furthermore, Jagged-1 regulates osteoclast differentiation directly and indirectly via the regulation of osteoprotegerin expression by human periodontal ligament cells [28]. In addition, analysis of the Reactome pathway of the RNA sequencing data indicates that Jagged-1 treated hDPs upregulated genes involved in the extracellular matrix (ECM) organisation [23]. Further, the ECM derived from the Jagged-1 activated Notch signalling pathway promotes mineralisation in stem cells isolated from apical papilla [13].

The ECMs are a three-dimensional network of protein/glycoprotein molecules that provide structural support for cells and tissues. It has been broadly known that the ECM orchestrate cell signalling, controls cellular functions, and regulates cell morphology [29]. The ECM derived from hDPSCs illustrates their competence in promoting mineralisation [30]. However, in-depth bioinformatics about how Jagged-1 regulates the extracellular matrix and induces osteogenic differentiation remains unexplored. To the best of our knowledge, this is the first report demonstrating the proteomic profiles of Jagged-1-induced mineralisation of hDPSc classified by the different cellular protein compartments.

## 2. Results

### 2.1. Characterisation of hDPSCs

**Figure 1:**
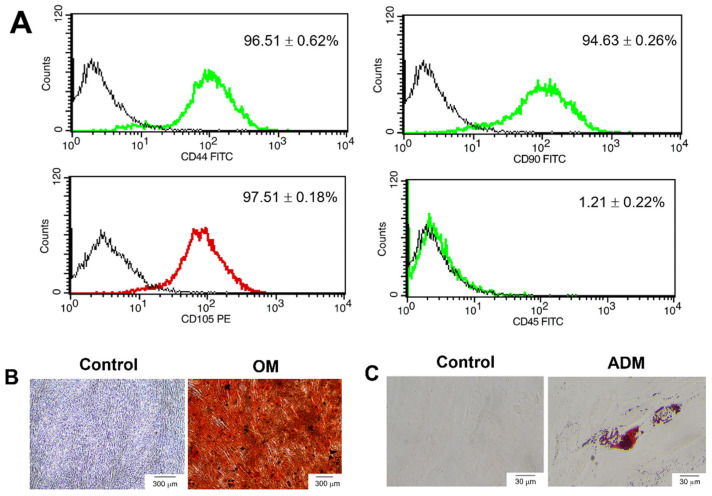
Characterisation of human dental pulp stem cells (hDPSCs). (**A**) The surface marker expression was analysed using flow cytometry. Multilineage differentiation potential to (**B**) osteogenic and (**C**) adipogenic lineage. Scale bars: 300 (**B**) and 30 (**C**) µm, respectively. The hDPSCs were cultured in an osteogenic differentiation medium (OM) and adipogenic differentiation medium (ADM). The mineral deposition was stained using Alizarin Red S (ARS) staining. The accumulation of lipid droplets in the cytoplasm was stained by Oil Red O staining.

The cells were characterised by the expression of cell surface antigens using flow cytometry (Figure 1A). hDPSCs were positively stained for the mesenchymal stem cell markers CD44 (96.51 ± 0.62%), CD90 (94.63 ± 0.26%) and CD105 (97.51 ± 0.18%) but negatively stained for the hematopoietic stem cell markers CD45 (1.21 ± 0.22%). hDPSCs could differentiate into osteogenic and adipogenic lineage upon culture in osteogenic and adipogenic induction, respectively. hDPSCs cultured in an osteogenic differentiation medium (OM) exhibited an increase in mineralisation compared with the undifferentiated control (Figure 1B). The accumulation of lipid droplets in the cytoplasm increased compared with the control (Figure 1C).

### 2.2. Jagged-1 Enhanced Osteogenic Differentiation of hDPSCs

**Figure 2:**
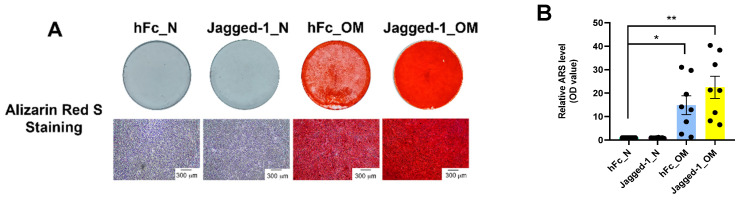
Indirect immobilisation Jagged-1 (Jagged-1) induced mineralisation in hDPSCs. On day 14, the mineralisation was examined by (**A**) ARS staining. (**B**) The relative value of the eluted ARS stain was measured at an absorbance of 570 nm. Asterisks indicated a statistically significant difference compared with the hFc_N. * *p* ≤ 0.05 and ** *p* ≤ 0.01.

After 14 days of differentiation, the mineralisation was determined by ARS staining. hDPSCs seeded on Jagged-1 coated surfaces cultured with OM showed nodular aggregates, and the ARS stain was more intense than other groups (Figure 2A). The eluted ARS stain indicated that mineral deposition increased significantly in Jagged-1 cultured in OM (Figure 2B) compared to the hFc control.

### 2.3. Subcellular Fractionation Showed the Existence of Matrisome Proteins

**Figure 3:**
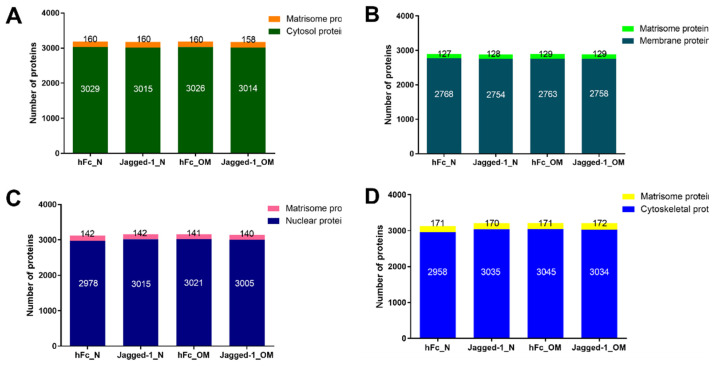
All cellular compartments exhibited the existence of matrisome proteins (**A**–**D**). The analyses were performed using the matrisome background list downloaded from http://matrisomeproject.mit.edu/other-resources/human-matrisome/ (accessed on 25 July 2022). Subsequently, the VLOOKUP function in Microsoft Excel (Microsoft 365, Microsoft, Washington, DC, USA) was applied to subtract the matrisome proteins from subcellular proteins.

The total protein in each compartment was approximately 3000 proteins under all culture conditions. The proteome analysis of all cellular compartments, including cytosol, membrane, nucleus, and cytoskeletal, demonstrated that matrisome proteins were found in all compartments, as shown in Figure 3A–D. The cytosol compartment contained 3014–3029 cytosolic proteins and 158–160 matrisome proteins (Figure 3A). The lowest number of total proteins was found in the membrane compartment. The membrane compartment consisted of 2754–2768 membranous proteins and 127–129 matrisome proteins (Figure 3B). The nuclear part illustrated 2978–3021 nuclear proteins and displayed 140–142 matrisome proteins (Figure 3C). Cytoskeletal proteins expressed 2958–3045 proteins for the cytoskeleton compartment along with 170–172 matrisome proteins (Figure 3D).

### 2.4. Each Cellular Compartment Exhibited a Different Amount of Matrisome Core and Matrisome-Associated Proteins

**Figure 4:**
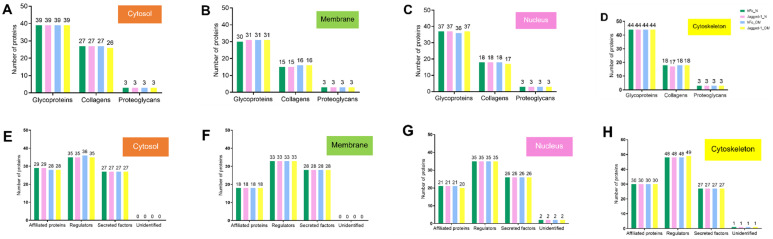
Core matrisome (**A**–**D**) and matrisome-associated proteins (**E**–**H**) in the cytosol, membrane, nucleus, and cytoskeleton compartments, respectively. The analyses were performed using the matrisome database downloaded from http://matrisomeproject.mit.edu/other-resources/human-matrisome/, accessed on 25 July 2022.

The matrisome was categorised into core matrisome and matrisome-associated proteins. The core matrisome comprised collagens, glycoproteins, and proteoglycans. The matrisome-associated proteins included ECM-affiliated proteins, ECM regulators, secreted factors, and unidentified proteins. The matrisome from all compartments and all treatment conditions were classified into core matrisome and matrisome-associated proteins according to the human matrisome database. The findings were described separately by the cell compartment. The results showed that the number of classified matrisome was slightly different among treatments. The cytosol had 68–69 core matrisome proteins (Figure 4A) and 90–91 matrisome-associated proteins (Figure 4B). The membrane compartment contained 48–50 core matrisomes (Figure 4C) and 79 matrisome-associated proteins (Figure 4D). For the nuclear part, there were 57–58 core matrisomes (Figure 4E) and 83–84 matrisome-associated proteins (Figure 4F). The cytoskeletal matrix proteins exhibited 64–65 core matrisomes (Figure 4G) and 106–107 matrisome-associate proteins (Figure 4H). Interestingly, the predominant core matrisome and matrisome-associated proteins in all compartments and all culture conditions were glycoproteins and ECM regulators, respectively.

### 2.5. A Venn Diagram Illustrated the Common and Uncommon Matrisome Proteins from Different Culture Conditions

**Figure 5:**
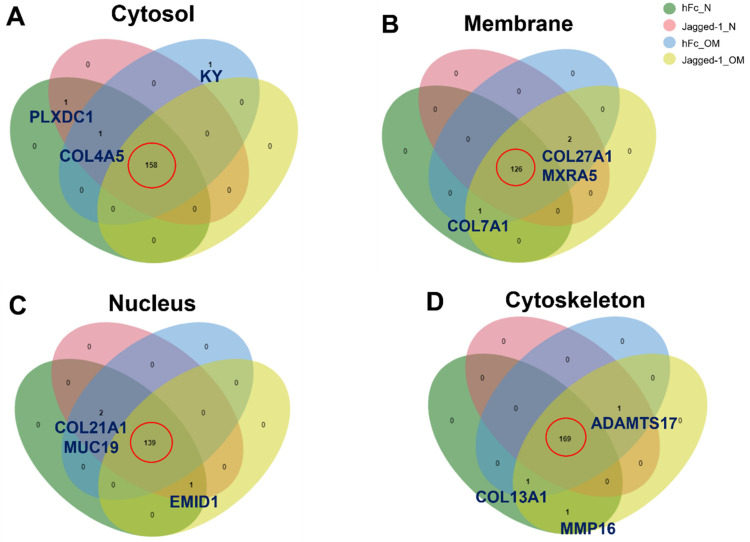
Venn diagram of common proteins and unique proteins presented in the (**A**) cytosol, (**B**) membrane, (**C**) nucleus, and (**D**) cytoskeleton compartment, respectively. The analyses were performed using an interactive Venn diagram viewer [31]. The red circle indicated the common matrisome proteins of the different culture conditions.

A Venn diagram was generated to separate the matrisome proteins of different culture conditions. For the cytosol, the chart showed 158 common matrisome proteins. The kyphoscoliosis peptidase (KY) protein was found only in the hFc_OM condition. Plexin domain-containing protein 1 (PLXDC1) was concomitantly found in cells treated with hFc_N and Jagged-1_N. Meanwhile, the collagen alpha-5 (IV) chain protein (COL4A5) was simultaneously expressed in hFc_N, hFc_OM, and Jagged-1_N culture conditions. Interestingly, we noticed that the aforementioned unique matrisome proteins were absent in the Jagged-1_OM culture condition (Figure 5A). For the membrane compartment, the number of intersecting matrisome proteins was 126. The collagen alpha-1 (VII) chain (COL7A1) was found in hFc_N, hFc_OM and Jagged-1_OM culture conditions. The other two uncommon proteins, collagen alpha-1 (XXVII) chain (COL27A1) and matrix-remodeling-associated protein 5 (MXRA5), were expressed in all culture conditions except hFc_N (Figure 5B). The nuclear compartment had 139 overlapped matrisome proteins. The collagen alpha-1 (XXI) chain (COL21A1) and mucin-19 (MUC19) were expressed under all culture conditions except Jagged-1_OM. In contrast, the EMI domain-containing protein 1 (EMID1) protein was recognised in all conditions except the hFc_OM condition (Figure 5C). The cytoskeletal compartment exhibited 169 shared matrisome proteins. The matrix metalloproteinase-16 (MMP16) was found in cells treated with hFc_N, and Jagged-1_OM. The collagen alpha-1 (XIII) chain (COL13A1) protein presented in all treatments except Jagged-1_N. A disintegrin and metalloproteinase with thrombospondin motifs 17 (ADAMTS17) was not found in the hFc_N culture condition. Remarkably, the Jagged-1_OM displayed all three uncommon matrisome proteins, MMP16, COL13A1, and ADAMTS17 (Figure 5D).

### 2.6. Metascape Gene List Analysis Reported the Relationship of Matrisome Proteins with Osteogenesis/Osteogenic Differentiation

**Figure 6:**
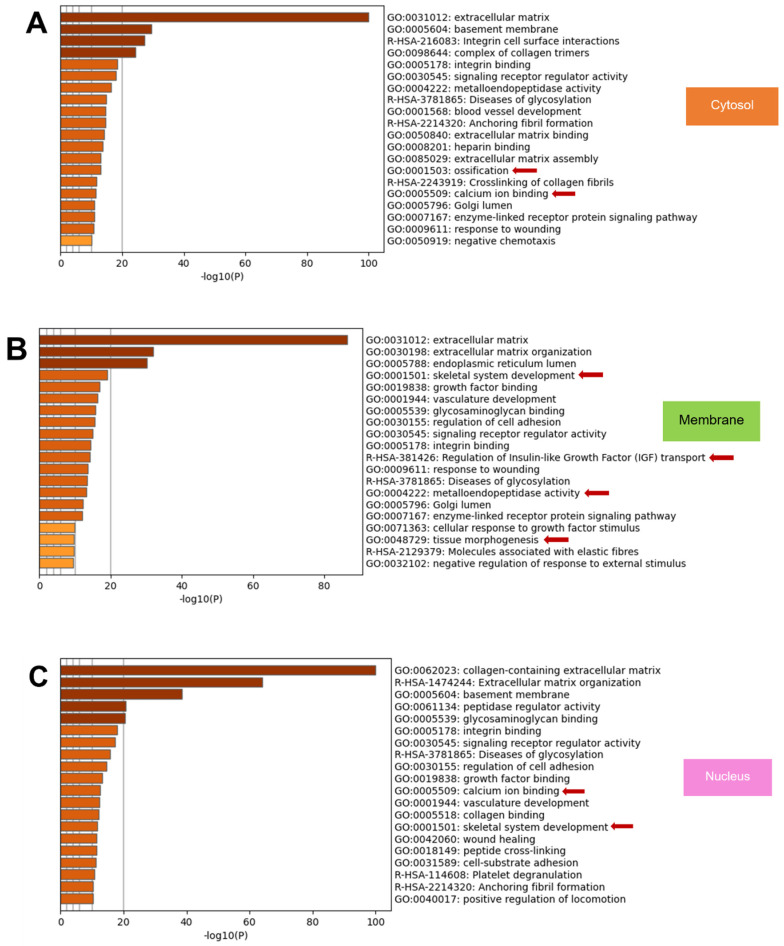

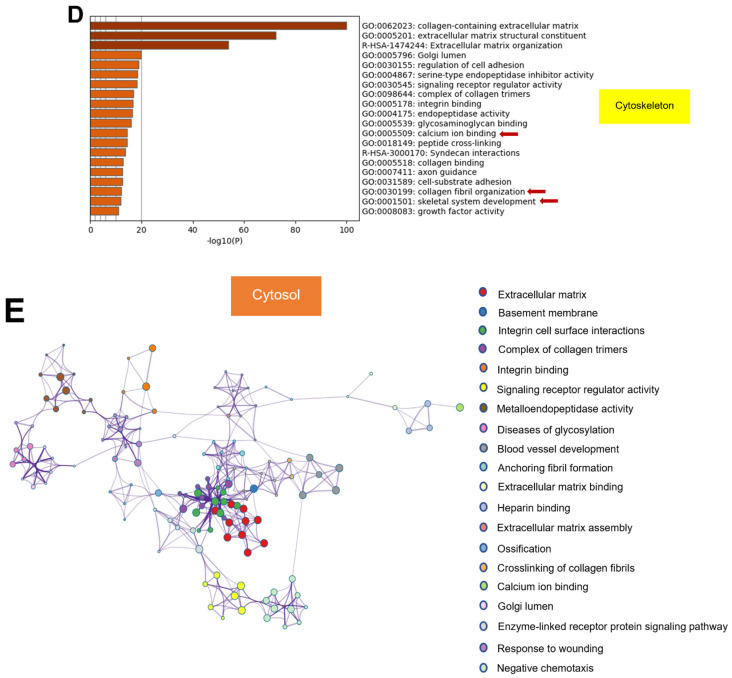

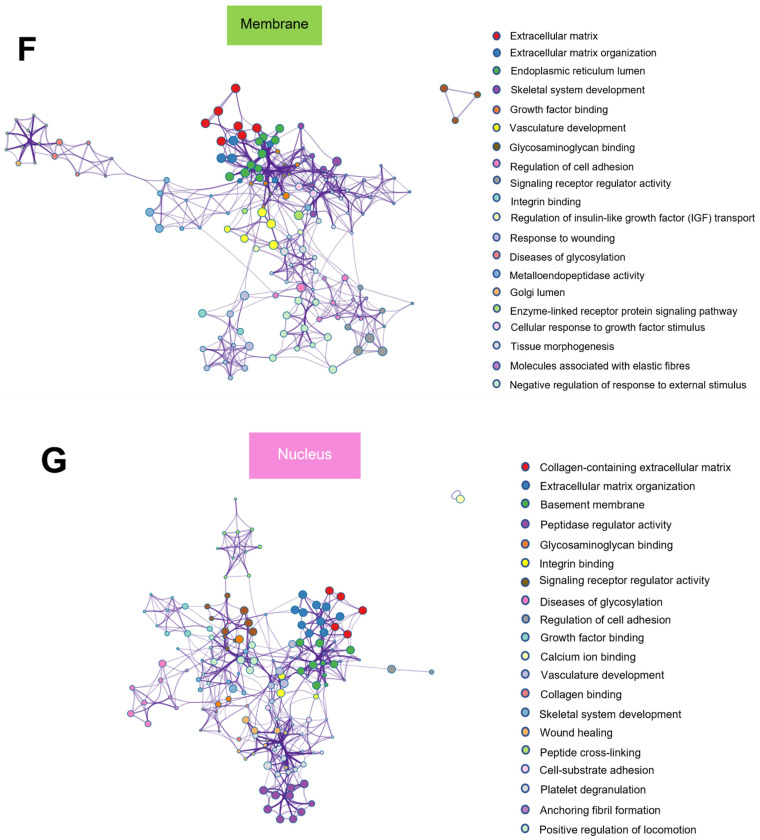

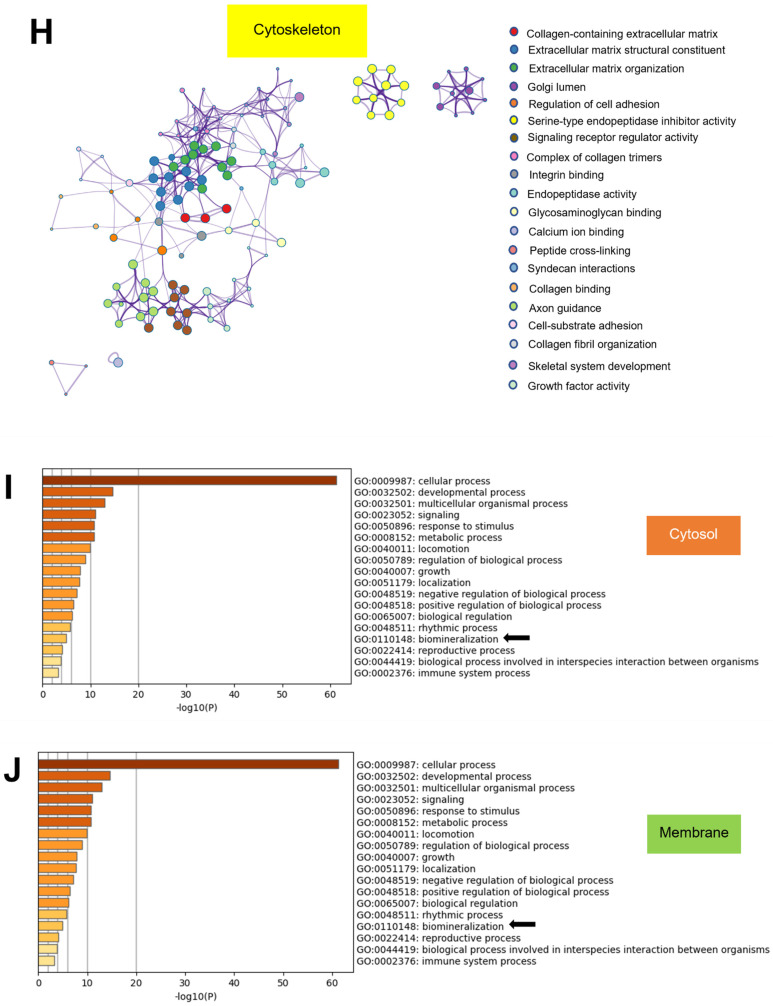

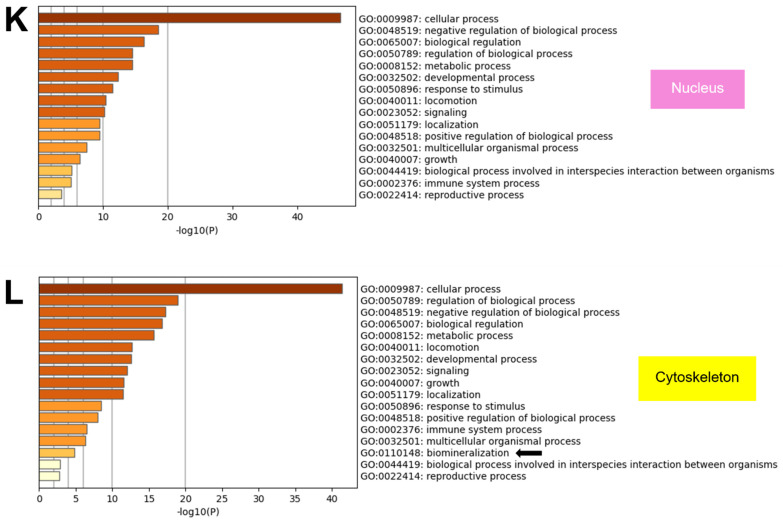
The metascape gene list analysis report demonstrated the bar graph of the top 20 enriched terms across input gene lists, colour by *p*-values, in (**A**) cytosol, (**B**) membrane, (**C**) nucleus, and (**D**) cytoskeleton compartment. The terms associated with osteogenesis/osteogenic differentiation were specified with red arrows. To show the relationship among terms, network plots by cluster identification (cluster ID) of the (**E**) cytosol, (**F**) membrane, (**G**) nucleus, and (**H**) cytoskeleton compartment were visualised using Cytoscape. The top-level gene ontology (GO) is categorised by the biological process classified by cellular compartment (**I**) cytosol, (**J**) membrane, (**K**) nucleus, and (**L**) cytoskeleton. Black arrows indicated the biomineralisation which participated in osteogenesis/osteogenic differentiation. The analyses were performed using Metascape [32].

The top 20 enriched terms across input gene lists coloured by *p*-values from all compartments represented several cell activities associated with osteogenesis/osteogenic differentiation (Figure 6). The cytosol matrisome proteins involve ossification and calcium ion binding (Figure 6A). The membrane proteins were related to the skeletal system development, regulation of insulin-like growth factor (IGF) transport, metalloendopeptidase activity and tissue morphogenesis (Figure 6B). The nuclear proteins are associated with calcium ion binding and skeletal system development (Figure 6C). The cytoskeletal component exhibited the proteins that regulated calcium ion binding, collagen fibril organisation, and skeletal system development (Figure 6D).

To further describe the relationships between the enriched terms, a subset of enriched terms was selected and rendered as a network plot. The network of enriched terms coloured by cluster identification (cluster ID) represented the relationship among clusters categorised by cellular compartment, including cytosol (Figure 6E), membrane (Figure 6F), nucleus (Figure 6G), and cytoskeleton (Figure 6H), respectively.

The top-level gene ontology (GO) categorised by the biological process showed that all compartments mainly expressed the proteins that functioned in the cellular process. However, the biomineralisation process, one of the fundamental processes in osteogenesis/osteogenic differentiation, was found in all compartments except the nuclear part (Figure 6I–L).

### 2.7. Protein–Protein Interaction Enrichment Analysis of Common Matrisome Proteins Demonstrated Distinctive Interaction among Different Cellular Compartments

**Figure 7:**
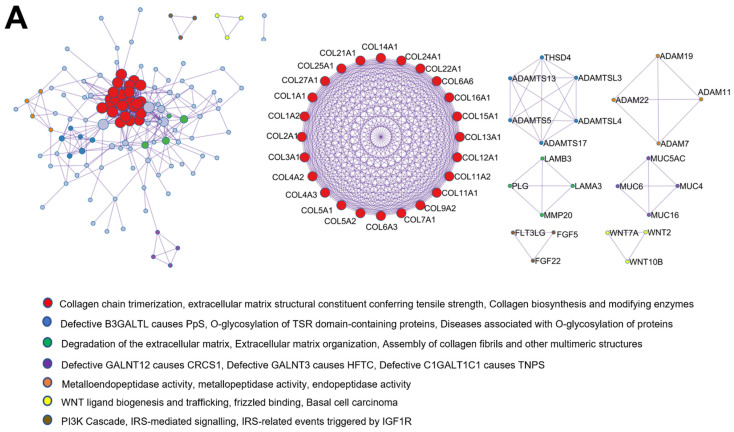

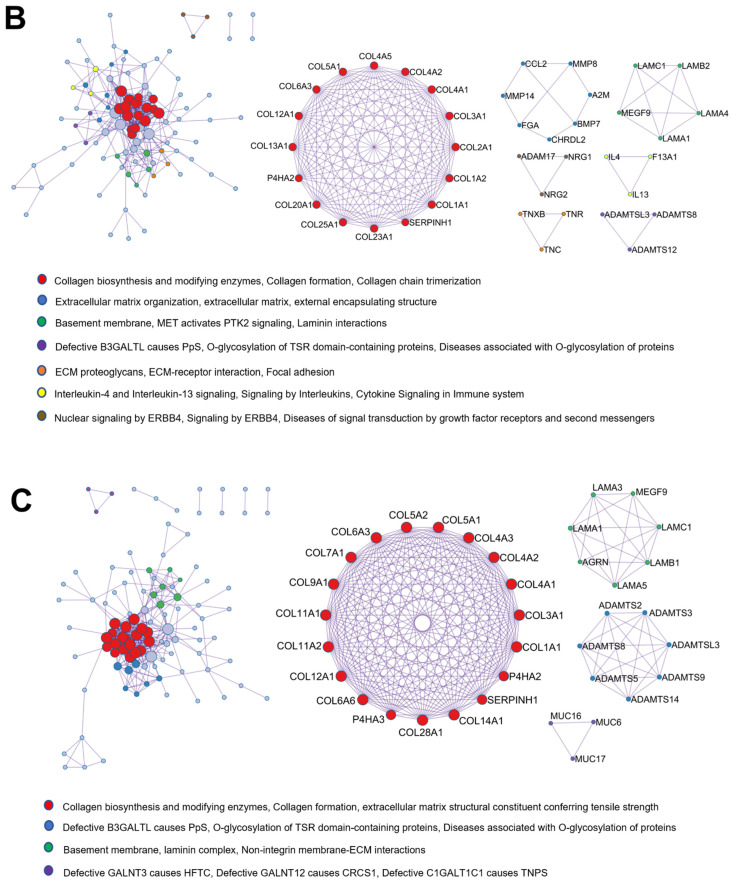

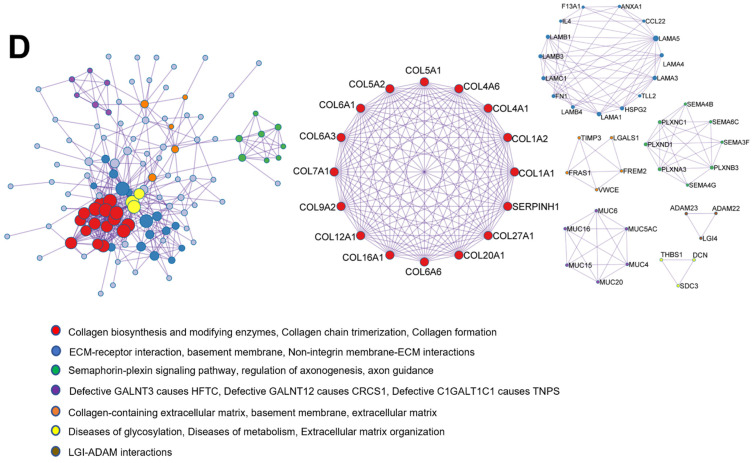
Schematic exhibited protein–protein interaction network and the Molecular Complex Detection (MCODE) components identified in the common matrisome gene lists of (**A**) cytosol, (**B**) membrane, (**C**) nucleus, and (**D**) cytoskeleton compartment, respectively. The analyses were performed using Metascape [32], and enrichment networks were generated using Cytoscape version 3.9.1 [33].

Metascape analysis was used to clarify the protein–protein interaction of matrisome proteins in each treatment group (Figure 7). The matrisome proteins of all compartments were mainly associated with ECM, external encapsulating structure, and collagen-containing ECM. However, the protein–protein interaction analysis revealed that each compartment had specific cellular functions in accordance with Molecular Complex Detection (MCODE) algorithm.

The cellular functions of matrisome proteins from the cytosol part primarily involved collagen chain trimerisation, extracellular matrix structural constituent conferring tensile strength, collagen biosynthesis, and modifying enzymes. In addition, the interaction of the proteins was associated with metalloendopeptidase activity, metallopeptidase activity, endopeptidase activity, WNT ligand biogenesis and trafficking, frizzled binding, and phosphatidylinositol 3-kinase (PI3K) Cascade (Figure 7A). The membrane matrisome protein–protein interactions were highly related to collagen biosynthesis and modifying enzymes, collagen formation, collagen chain trimerisation, basement membrane, laminin interactions, ECM proteoglycans, ECM–receptor interaction, and focal adhesion (Figure 7B). The nuclear matrisome proteins were associated with collagen biosynthesis and modifying enzymes, collagen formation, extracellular matrix structural constituent conferring tensile strength, basement membrane, laminin complex, and non-integrin membrane–ECM interactions (Figure 7C). The protein–protein interaction of the matrisome in the cytoskeleton compartment was principally linked to collagen biosynthesis and modifying enzymes, collagen chain trimerisation, collagen formation, semaphorin–plexin signalling pathway, regulation of axonogenesis, axon guidance, collagen-containing extracellular matrix, and basement membrane (Figure 7D).

### 2.8. GO from Different Culture Conditions in Each Cellular Component Displayed Several Unique Gene Ontologies

**Figure 8:**
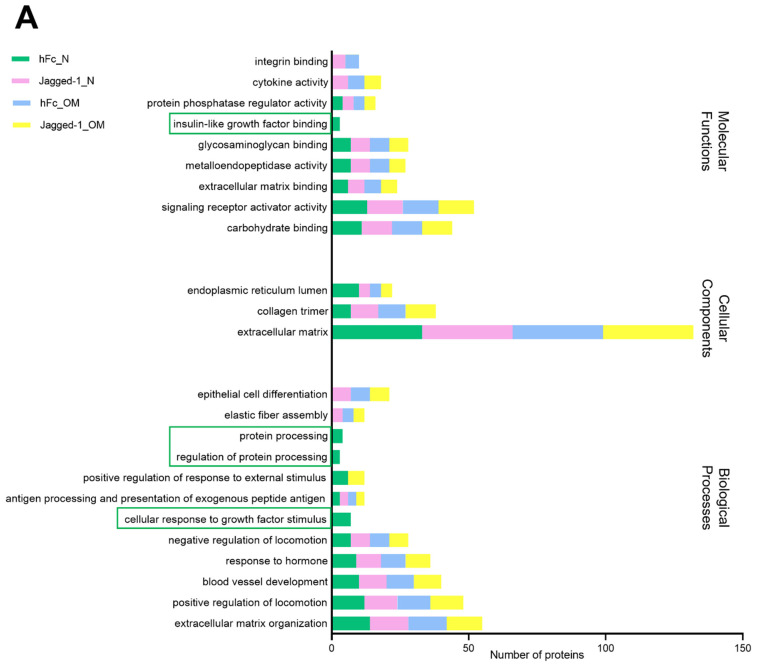

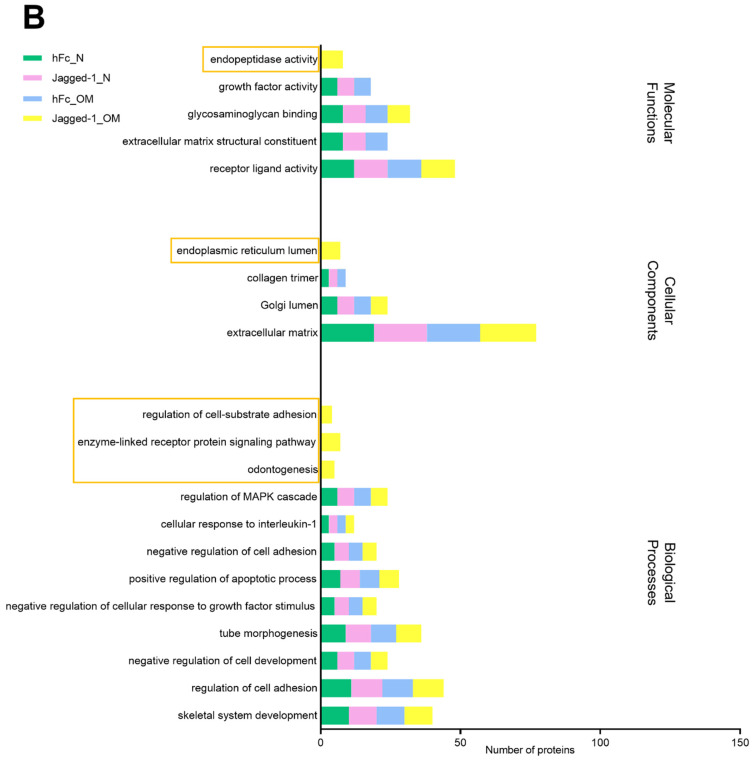

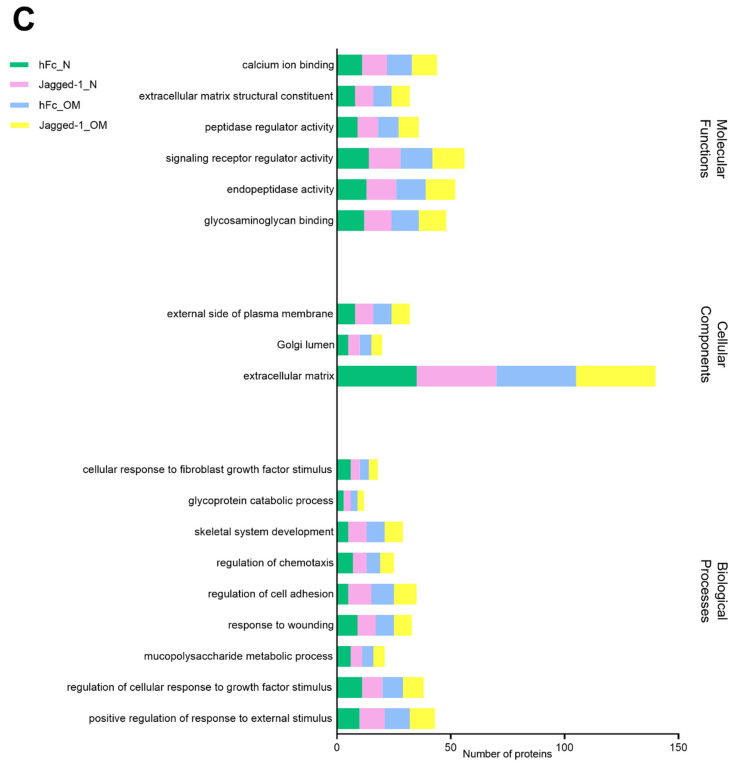

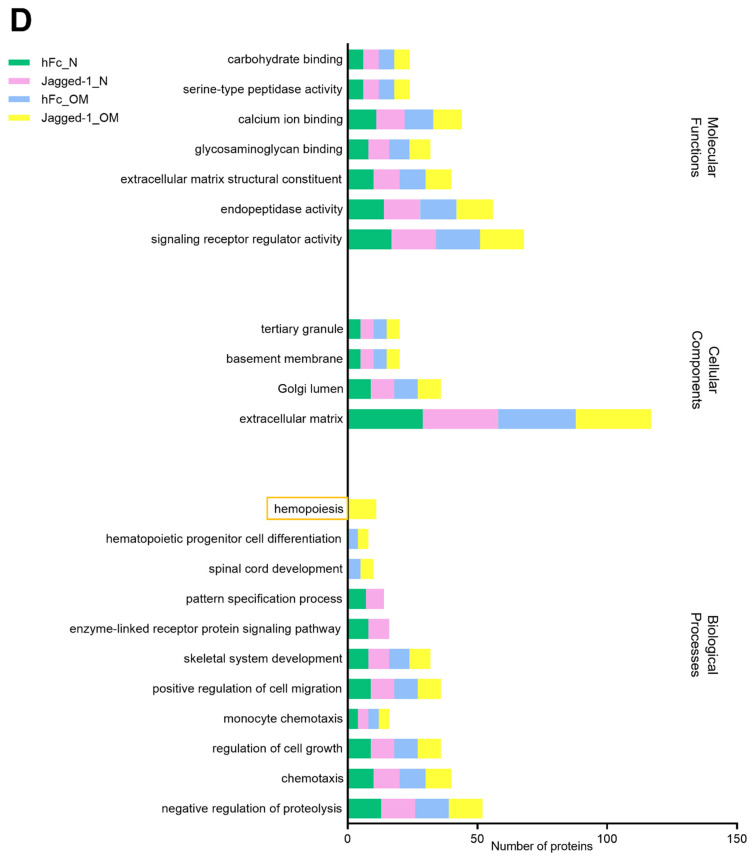
Bar graphs showed the gene ontology in biological processes, cellular components, and molecular functions of (**A**) the cytosol, (**B**) membrane, (**C**) the nucleus, and (**D**) the cytoskeleton compartment, respectively. Unique proteins were specified in a rectangular shape. The analyses were performed using Metascape [32].

For each culture condition, matrisome gene list, pathway and process enrichment analysis had been carried out with the following GO sources: GO Biological Processes, GO Cellular Components, GO Molecular Functions, KEGG Pathway, Reactome Gene Sets, and PANTHER Pathway. The top 20 GO of all compartments were mainly involved in biological processes, cellular components, and molecular functions. The unique ontology has been found in the components of the cytosol, membrane, and cytoskeleton. In the cytosol component, the hFc_N condition showed four unique ontologies: IGF binding, protein processing, regulation of protein processing, and cell response to growth factor stimulus (Figure 8A). The membrane compartment of the Jagged-1_OM condition showed five unique ontologies: endopeptidase activity, endoplasmic reticulum lumen, regulation of cell–substrate adhesion and enzyme-linked receptor protein signalling pathway, and odontogenesis (Figure 8B). The nuclear matrisome proteins from different culture conditions did not present a unique gene ontology. In the cytoskeleton compartment, Jagged-1_OM had a unique ontology that participated in hemopoiesis (Figure 8D).

## 3. Discussion

The present study demonstrated that Jagged-1 potentially induced osteogenic differentiation in hDPSCs as shown in previous studies [23,24]. Furthermore, the previous data indicate the Reactome pathway relationship between Jagged-1 and ECM on osteogenic differentiation of hDPs [23]. Therefore, our objective was to discover the intracellular mechanism(s) related to these two components that regulated osteogenic differentiation. In this study, hDPSCs cultured in OM on a Jagged-1 coated surface were used. Protein was extracted from cells after culture for 14 days using specific kits that separated subcellular components into cytosol, membrane, nucleus, and cytoskeleton. Subsequently, all fractions were analysed by liquid chromatography–tandem mass spectrometry (LC-MS). Thus, the advantage of this study is providing a specific proteomic profile of each subcellular component.

The Venn diagram of the entire proteome (Figure S1) and matrisome proteins in all components (Figure S2), separated by culture condition, did not reveal any specific protein related to osteogenic differentiation. Jagged-1_OM culture condition presented only one unique protein, Protein dopey-2 (DOP1B), which generally functions on regulating membrane trafficking of cargo proteins [34] (Figure S1). Although the membrane trafficking process does not directly influence cell differentiation, the bidirectional effects of membrane trafficking and differentiation can control cell fate. The membrane trafficking impacts signalling pathways by controlling the activity of ligands and receptors [35]. Membrane trafficking regulates cell differentiation by sending and receiving signals through endocytosis, exocytosis, and extracellular vesicles [36,37,38]. Notch signalling is also regulated by endocytosis and endosomal trafficking [39]. Therefore, we speculate that DOP1B might be responsible for assisting the membrane trafficking of our transmembrane Notch ligand, Jagged-1.

The information from the whole proteome might provide nonspecific data; therefore, subcellular proteome profiles were further analysed. Here, we demonstrate the basic quantitative information of matrisome proteins in all cellular compartments and all culture conditions. Although the ECM are outside the cells, matrisome proteins exist in all cellular compartments. The human matrisome comprises 1068 proteins classified into 274 core matrisomes, 753 matrisome-associated proteins, and 41 unidentified proteins [40]. The matrisome proteins in each subcellular compartment displayed 12-16% of the human matrisome (Figure 3). However, there were overlapped matrisome proteins in each compartment. Therefore, the whole cells exhibited approximately 36% of human matrisome (Figure S2). The existence of matrisome proteins in all cellular components emphasises the interactions between cells and ECM microenvironments on essential biological processes, which are mediated by the matrisome proteins [41].

The matrisome proteins of each culture condition In all compartments were separated by a Venn diagram. Jagged-1_OM in all subcellular components did not present a unique matrisome protein. However, there were several unique overlapped proteins that Jagged-1_OM shared with other culture conditions, including COL27A1, MXRA5, COL7A1, EMID1, ADAMTS17, COL13A1, and MMP16. Some aforementioned proteins facilitate cell differentiation, as reported in previous studies. For example, COL27A1 involves bone morphogenesis, chondrocyte differentiation, and cartilage development [42]. A mutation in *COL27A1* is identified as the cause of Steel syndrome which observed symptoms consistent with systemic bone disease [43]. The MXRA family comprises three genes (*MXRA5, MXRA7,* and *MXRA8*), which participate in cell adhesion and matrix remodelling [44]. MXRA5 is matrix remodelling associated five that is associated with chondrogenic differentiation [45]. MXRA5 is also identified as a fibrosis inhibitor and transforming growth factor-beta (TGF-β) signalling regulator [46]. TGF-β signalling appears to be involved primarily in the early phase of osteogenesis and allows osteoprogenitor cells to differentiate into immature osteoblasts [47]. Moreover, TGF-βs and bone morphogenetic proteins (BMPs) transduce signals to control mesenchymal stem cell differentiation during skeletal development and bone formation. The signal transduction can occur via the canonical Smad-dependent signalling pathway and the noncanonical Smad-independent signalling pathway. The interaction between TGF-β and receptor leads to the suppressor of mothers against decapentaplegic (SMAD) 2/3 phosphorylation for the Smad-dependent signalling pathway. Phosphorylated SMAD2/3 binds to the SMAD4 protein and migrates to the nucleus to control the expression of runt-related transcription factor (Runx2) [48]. The core-binding factor subunit alpha-1 (Cbfa1)/Runx2, a bone transcription factor, is a critical gene that encodes proteins related to the osteogenic differentiation process from mesenchymal precursor cells. Runx2-deficient mice exhibit an arrest in osteoblast differentiation, resulting in a complete lack of ossification [49,50]. COL7A1 promotes three-dimensional osteogenic differentiation of bone marrow mesenchymal stem cells (BMSCs) [51]. MMP16 is a protein found in osteoblasts and osteocytes. MMP16 separates ECM proteins, such as type I fibrillar collagen, and promotes bone growth and development [52]. *MMP16* upregulation is found in BMSCs after 14 days of osteogenic differentiation medium [53]. Interestingly, in the present study, the proteins related to cell differentiation were mainly obtained from the membrane compartment. These results imply that Jagged-1, a transmembrane Notch ligand, promotes the expression of ECM proteins related to cell differentiation and can transmit the signals of the membrane trafficking cargo proteins to the cell to activate similar proteins to drive the differentiation process.

Although some unique proteins showed their relationship to cell differentiation and osteogenesis, we further analysed the data of the common matrisome proteins of each compartment (Figure 6). The metascape gene list analysis demonstrated several enriched terms related to osteogenesis/osteogenic differentiation, such as ossification and calcium ion binding in all subcellular compartments (Figure 6A–D). We noticed that the membrane compartment had shown different enriched terms: the regulation of IGF transportation, metalloendopeptidase activity, and tissue morphogenesis. These enriched terms of the membrane compartment may facilitate the other compartment proteins in promoting osteogenesis/osteogenic differentiation. IGFs (IGF-1 and IGF-2) control and stimulate stem cell differentiation in vitro and in vivo [54,55]. IGF-1 is a key regulator that controls growth and differentiation in numerous cell types. For example, IGFs can promote endothelial differentiation of embryonic stem cells [56]. IGF-1 can promote osteogenic differentiation of various dental tissues, including hDPSCs [57], apical papilla [58], and periodontal ligament stem cells (PDLSCs) [59]. As for hDPSCs, IGF-1 promotes osteoblast differentiation by activating the mammalian target of rapamycin (mTOR) through the PI3K/Akt (Protein Kinase B) pathway [60,61]. In addition, IGF-I promotes the proliferation and odonto/osteogenic differentiation of hDPSCs by activating the Mitogen-activated protein kinase (MAPK) signalling pathway [57]. Exogenous expression of IGF-2 enhances BMP-9 and induces the osteogenic marker alkaline phosphatase (ALP), osteocalcin (OCN) and osteopontin (OPN) in mesenchymal stem cells (MSCs) [62]. For metalloendopeptidase, it is an endopeptidase which uses a metal such as zinc in the catalytic mechanism. A subcategory of metalloendopeptidase, which plays a crucial role in bone remodelling, is matrix metalloproteinases (MMP). The MMPs are enzymes responsible for the proteolytic degradation of ECM components such as collagen [63]. A previous study reported that MMP-1 accelerated the osteogenic differentiation of bone marrow stem cells (BMSC) through the Jun kinases (JNK) and extracellular signal-regulated kinase (ERK) pathways. Knockdown of MMP-1 decreased migration, proliferation, and osteogenic differentiation of BMSCs [64]. The study of ECM derived from human bone marrow mesenchymal stem cells induced osteogenic differentiation within porous chitosan/gelatin scaffolds revealed a significant increase in *MMP1, MMP9, MMP11* and *MMP16* on day 14 of osteogenic differentiation detected by polymerase chain reaction (PCR) arrays [53]. Taken together, the membrane matrisome proteins (1) participate in osteogenesis/osteogenic differentiation by increasing the possibility of IGFs transportation into the cells, (2) enhance the catalytic mechanism of bone remodelling, and (3) stimulate tissue regeneration, and simultaneously modify tissue morphogenesis.

The top-level GO categorised by the biological process (Figure 6I-L) showed that all compartments except the nucleus expressed the proteins involved in biomineralisation. The absence of protein related to biomineralisation in the nuclear compartment can imply that the modulation of protein that functioned on the biomineralisation process occurs outside the nucleus. However, the nuclear compartment is crucial for biomineralisation because the nucleus GO shows essential roles in overall biological regulation and developmental process.

The protein–protein interaction network and the MCODE components identified in the common matrisome gene lists (Figure 7) elucidate that collagens are the most abundant components. Correspondingly, the protein–protein interaction of hDPSCs matrisome proteins has highlighted the network of interaction between the synthesis of collagen, integrin membranes, elastic fibres, and laminin, as well as the activation of focal adhesion MET receptor and the interaction of the neural cell adhesion molecule (NCAM) [30]. All subcellular components participated mainly in collagen biosynthesis and expressed type I collagen. Collagen biosynthesis takes place both intra- and extra-cellularly. Different types of collagens may undergo different post-translational modifications [65]. Type I collagen is the major structural protein in the human body, typically found in bone and skin [66]. Adding solubilised type I collagen to the osteogenic medium facilitates osteogenic differentiation and mineralised bone matrix of rat MSCs [67]. Hence, the presence of numerous type I collagen in the matrisome proteins shows more advantages to osteogenesis/osteogenic differentiation because there are abundant substrates for mineralisation.

The top 20 GO of all compartments were mainly involved in biological processes, cellular components, and molecular functions (Figure 8). The unique ontology of each culture condition has been found in all components except the nucleus. In the cytosol, the hFc_N condition showed four unique ontologies: IGF binding, protein processing, regulation of protein processing, and cellular response to growth factor stimulus. It might be inferred that the hFc_N culture condition was the only group that had cellular responses that occurred directly through IGF-binding proteins (IGFBP) in the cytosol area. Previous studies suggest that IGFBPs generally bind to IGFs with an affinity superior to that of the IGF-1 receptor. This phenomenon can inhibit IGF signalling by ligand sequestration [68,69,70]. Therefore, the cell differentiation detected by mineralisation assay was not found in hFc_N because the IGFBPs/IGF complexes blocked the IGF-1 receptor-binding region of IGF-1. On the contrary, only Jagged-1_OM of the cytoskeletal part exhibited a unique matrisome protein related to hemopoiesis. This may indicate the co-evidence of osteogenesis and hematopoiesis. The interplay between osteogenesis and hematopoiesis has been reported [71]. Osteogenic cells have been shown to be crucial in expanding and conserving hematopoietic stem/progenitor cells [72]. The gene in the CBF family encrypts an essential group of heterodimeric transcription factors that act as the main regulators of developmental gene expression for both hematopoiesis and osteogenesis. Acute myelogenous leukaemia (AML) (also known as CBFA2) encodes a DNA-binding Runt domain of the heterodimeric core-binding factor. Cbfa2-deficient mice lack erythropoiesis and myelopoiesis [73]. Thus, this co-evidence suggests that Jagged-1_OM can be applied in tissue/organ regeneration to stimulate osteogenesis and hematopoiesis. Although the cytosol and cytoskeleton compartments demonstrated several specific gene ontologies, the membrane compartment displayed more interesting gene ontologies, especially in osteogenesis. The spotlight is shaded to the membrane matrisome protein once again. The gene ontology of Jagged-1_OM membrane matrisome proteins demonstrated the correlation with endopeptidase activity, endoplasmic reticulum lumen, regulation of cell–substrate adhesion, enzyme-linked receptor protein signalling pathway, and odontogenesis. Together, the results suggested that the Jagged-1_OM membrane matrisome proteins regulate membrane functions and play a pivotal role in odontogenesis.

In summary, we address the question of how Jagged-1 regulates ECM and induces osteogenic differentiation in terms of cellular evidence. Subcellular fractionation provides more specific data on the expression of ECM proteins in each compartment. In-depth bioinformatics information revealed the interplay of Jagged-1 and ECM on osteogenic differentiation. We discovered that (1) Jagged-1 stimulated the expression of both direct and indirect proteins associated with osteogenic differentiation, (2) matrisome proteins in all cellular components participated in osteogenic differentiation, and (3) the matrisome proteins derived from Jagged-1_OM of the membrane compartment elucidated the role in odontogenesis. However, further studies on the ECM itself, such as modification of the ECM protein or remodelling of the ECM after Jagged-1 stimulation, would provide more information on the bidirectional effects between Jagged-1 and the ECM on osteogenic differentiation.

## 4. Materials and Methods

### 4.1. hDPSCs Isolation and Culture

The protocol was approved by the Human Research Ethics Committee of Chulalongkorn University (approval no. 088/2021) and performed following the guidelines of the Biosafety Committee of the Faculty of Dentistry, Chulalongkorn University. Briefly, the impacted third molars, scheduled to be extracted according to the patient’s treatment plan, were smashed to access the dental pulp tissues. The pulp tissues were chopped and placed in 35 mm tissue culture plates for cell explantation. The growth medium consisted of Dulbecco’s Modified Eagle Medium (DMEM, cat. no. 11960, Gibco, Waltham, MA, USA) containing 10% fetal bovine serum (FBS, cat. no. 10270, Gibco, Waltham, MA, USA), 2 mM L-glutamine (GlutaMAX-1, cat. no. 35050, Gibco, Waltham, MA, USA), 100 unit/mL penicillin, 100 μg/mL streptomycin, and 250 ng/mL amphotericin B (Antibiotic–Antimycotic, cat. no. 15240, Gibco, Waltham, MA, USA). Cells were grown in a humidified incubator with 5% CO_2_ at 37 °C. The growth medium was changed every alternate day. Cells passage 3rd–7th was used for subsequent assays. Cells from at least three different donors were employed in all experiments.

### 4.2. Jagged-1 Immobilisation

Indirect immobilised Jagged-1 was coated on the surface of the tissue culture plate, as previously described [23]. Briefly, the 24-well-plates were used for the mineralisation assay, and 120 mm Petri dishes were utilised for the protein extraction. The tissue culture plate was coated overnight with 50 μg/mL recombinant protein G (cat. no. 101201, Invitrogen, Rockford, IL, USA). The incubated plates were washed with sterile phosphate-buffered saline (PBS). The culture plates were coated with 10 mg/mL bovine serum albumin (cat. no. A9418, Sigma-Aldrich, St. Louis, MO, USA) for 2 h and subsequently washed with PBS. The final coating step was performed by incubating 10 nM of human IgG Fc fragment (hFc, cat. no. 009000008, Jackson Immuno Research Labs, West Grove, PA, USA) and 10 nM rhJagged1/Fc recombinant protein (cat. no. 1277-JG, R&D Systems, Minneapolis, MN, USA) for 2 h. All procedures were performed at room temperature.

### 4.3. Cell Preparation

Cells were seeded on hFc/ Jagged1 immobilised tissue culture surfaces. Cells cultured in each type of coated plate were divided into 2 culture conditions. In the first condition, cells were cultured in the growth medium for 7 days and subsequently changed to a growth medium supplement with 50 μg/mL ascorbic acid (which was stated as “N medium”) for another 7 days. Regarding the second condition, cells were grown in an OM consisting of a growth medium supplemented with 50 mg/mL ascorbic acid, 5 mM beta-glycerophosphate, and 250 μM dexamethasone. The osteogenic differentiation period was 14 days. The medium was changed every other day.

### 4.4. Mineralisation Assay

Mineral deposition was examined using an ARS staining assay according to the previously described [74,75]. Cells were fixed with 4% paraformaldehyde and rinsed with deionised water. The ARS solution at 1% *w/v* concentration was used to stain the cells for 3 min. The excessive colour from ARS staining was gently washed with deionised water. The culture plates were then flipped and dried overnight. Microscopic images of the mineralisation area were taken with a Microscope ECLIPSE Ts2, Nikon-DS-Fi3, Minato-ku, Tokyo, Japan). Consequently, the staining was eluted using 10% cetylpyridium chloride monohydrate in 10 mM sodium phosphate (Sigma Aldrich, St Louis, MO, USA). The absorbance at 570 nm was examined using a microplate reader (Biotek ELX800, Santa Clara, CA, USA).

### 4.5. Subcellular Extraction

Protein extraction was performed using cells from three different donors. After 14 days of culture, cells were extracted by ProteoExtract^®^Subcellular Proteome Extraction Kit (Cat. no. 539790, Calbiochem^®^, San Diego, CA, USA). The extraction steps were performed following the manufacturer’s protocol. The sequence of protein fractions obtained from the kit was ordered from cytosolic protein, membrane protein, nuclear protein, and cytoskeletal protein, respectively.

### 4.6. Sample Preparation for Shotgun Proteomics

Each cellular compartment was crushed to powder in liquid nitrogen. One hundred milligrams of the powder was mixed with 0.5% Sodium Dodecyl Sulfate (SDS), vortexed for 1 h, and subsequently centrifuged at 10,000× *g* for 15 min. Cold acetone was mixed with the supernatant from the previous step and incubated at −20 °C for the night. The mixture was centrifuged at 10,000× *g* for 15 min. The pellet was dried and kept at −80 °C. Samples were dissolved with 0.1% SDS and protein concentration was evaluated using the Lowry method. Bovine serum albumin was used as a standard protein.

### 4.7. Sample Preparation

To reduce the disulfide bond, proteins were added with 10 mM dithiothreitol in 10 mM ammonium bicarbonate. Alkylation was applied using 30 mM iodoacetamide in 10 mM ammonium bicarbonate to block the disulfide molecules rebonding. The samples were further digested by porcine trypsin in a 1:20 ratio for 16 h at 37 °C. The drying process was performed by a speed vacuum concentrator. Subsequently, protein resuspension was performed using 0.1% formic acid. The resuspended samples were analysed by nano-liquid chromatography–tandem mass spectrometry (nanoLC-MS/MS).

### 4.8. Liquid Chromatography–Tandem Mass Spectrometry (LC-MS)

The tryptic peptide samples were prepared for injection to an Ultimate3000 Nano/Capillary LC System (Thermo Scientific, Oxford, UK) coupled to an HCTUltra LC-MS system (Bruker Daltonics Ltd.; Hamburg, Germany) equipped with a Nano-captive spray ion source as described in a previous study [76]. The LC-MS analysis of each sample was performed in triplicate.

### 4.9. Bioinformatics and Data Analysis

LC-MS data were performed using DecyderMS [77,78] using the Homo sapiens protein database from UNIPROT (December 2021). Searches were performed with a maximum of three missed cleavages, carbamidomethylation of Cys as a fixed modification, and oxidation of Met as variable modifications. The samples expressed the protein level as a log 2 value. The visualisation and statistical analyses were conducted using the MultiExperiment Viewer (MeV) in the TM4 suite software [79]. Proteomics data were sorted and rearranged using Microsoft Excel (Microsoft 365, Microsoft, Washington, DC, USA). The genes encoding the human matrisome and categorical matrisome were downloaded from http://matrisomeproject.mit.edu/other-resources/human-matrisome/, accessed on 25 July 2022 to specify the matrisome proteins from the analysed proteome profiles. Data were further processed using a Venn diagram [31] to show differences between protein lists originating from different treatments. Pathway and process enrichment analysis and protein–protein interaction enrichment analysis were performed using Metascape [http://metascape.org, accessed on 25 July 2022] [32]. For each matrisome list, pathway and process enrichment analysis was carried out with the following ontology sources: GO biological processes, GO cellular components, GO molecular functions, Reactome gene sets, KEGG pathway, and Panther pathway. Protein–protein interaction enrichment analysis was carried out with the Metascape databases. The resulting network contains the subset of proteins that form physical interactions with at least one other member in the protein list. If the network contains between 3 and 500 proteins, the MCODE algorithm was applied to identify closely connected network components. Cytoscape version 3.9.1 [33] was used to create the layout of the protein–protein interaction network.

### 4.10. Statistical Analysis

Statistical analysis of the eluted ARS stain was performed using the Kruskal–Wallis test, followed by multiple comparisons of the Dunn test using GraphPad Prism version 8.0.1. Statistical significance was determined at *p* ≤ 0.05.

## 5. Conclusions

In conclusion, we provide a fundamental proteomic profile of hDPSCs cultured on surfaces coated with hFc and Jagged-1 in normal and osteogenic differentiation environments. Furthermore, the analysis of subcellular components presents novel information on the osteogenic differentiation of hDPSCs. The indirect immobilisation of Jagged-1 stimulates the expression of membrane trafficking protein (DOP1B), which may indirectly enhance osteogenic differentiation. Furthermore, Jagged-1_OM expresses several proteins associated with osteogenic differentiation (COL27A1, MXRA5, COL7A1, and MMP16). Several gene ontologies indicate that the matrisome proteins in all subcellular compartments participate in bone formation, e.g., calcium ion binding, skeletal system development, and biomineralisation. Furthermore, the Jagged-1_OM membrane matrisome proteins demonstrate an outstanding function in odontogenesis. Taken together, indirect immobilisation of Jagged-1 improves matrisome proteins associated with odonto/osteogenic differentiation of hDPSCs.

## Data Availability

Any data or material supporting this study’s findings can be made available by the corresponding author upon request.

## Supplementary Materials

The supporting information can be downloaded at: https://www.mdpi.com/article/10.3390/ijms232213897/s1.

## Abbreviations

ADAMTS17	A disintegrin and metalloproteinase with thrombospondin motifs 17
ADM	Adipogenic differentiation medium
Akt	Protein Kinase B
ALP	Alkaline phosphatase
AML	Acute myelogenous leukaemia
ARS	Alizarin Red S
BMPs	Bone morphogenetic proteins
BMSCs	Bone marrow stem cells
Cbfa1	Core-binding factor subunit alpha-1
Cluster ID	Cluster identification
COL7A1	Collagen alpha-1 (VII) chain
COL13A1	Collagen alpha-1 (XIII) chain
COL21A1	Collagen alpha-1 (XXI) chain
COL27A1	Collagen alpha-1 (XXVII) chain
COL4A5	Collagen alpha-5 (IV) chain
DOP1B	Protein dopey-2
ECM	Extracellular matrix
EMID1	EMI domain-containing protein 1
ERK	Extracellular signal-regulated kinase
GO	Gene ontology
hDPs	Human dental pulp cells
hDPSCs	Human dental pulp stem cells
hFc	Human IgG Fc fragment
IGF	Insulin-like growth factor
IGFBPs	Insulin-like growth factor-binding proteins
Jagged-1	Indirect immobilised Jagged-1
JNK	Jun kinases
KY	Kyphoscoliosis peptidase
LC-MS	Liquid Chromatography–Tandem Mass Spectrometry
MAPK	Mitogen-activated protein kinase
MCODE	Molecular Complex Detection
MeV	MultiExperiment Viewer
MMP16	Matrix metalloproteinase-16
MSCs	Mesenchymal stem cells
mTOR	Mammalian target of rapamycin
MUC19	Mucin-19
MXRA5	Matrix-remodeling-associated protein 5
NanoLC-MS/MS	Nano-liquid chromatography–tandem mass spectrometry
NCAM	Neural cell adhesion molecule
NICD	Notch intracellular domain
OCN	Osteocalcin
OM	Osteogenic differentiation medium
OPN	Osteopontin
PBS	Phosphate-buffered saline
PCR	Polymerase chain reaction
PDLSCs	Periodontal ligament stem cells
PI3K	Phosphatidylinositol 3-kinase
PLXDC1	Plexin domain-containing protein 1
PPI	Protein–protein interaction
Runx2	Runt-related transcription factor 2
SDS	Sodium Dodecyl Sulfate
SMAD	Suppressor of mothers against decapentaplegic
TGF-β	Transforming growth factor-beta
