# Supplementary material for: Indirect Immobilised Jagged-1 Enhances Matrisome Proteins Associated with Osteogenic Differentiation of Human Dental Pulp Stem Cells: A Proteomic Study
Source: Int J Mol Sci. 2022 Nov 11;23(22):13897. doi: 10.3390/ijms232213897 (PMC9694941; doi:10.3390/ijms232213897)
Supplement: Supplementary file 1 [file ijms-23-13897-s001.zip › ijms-2001246-supplementary.pdf]

Supplementary

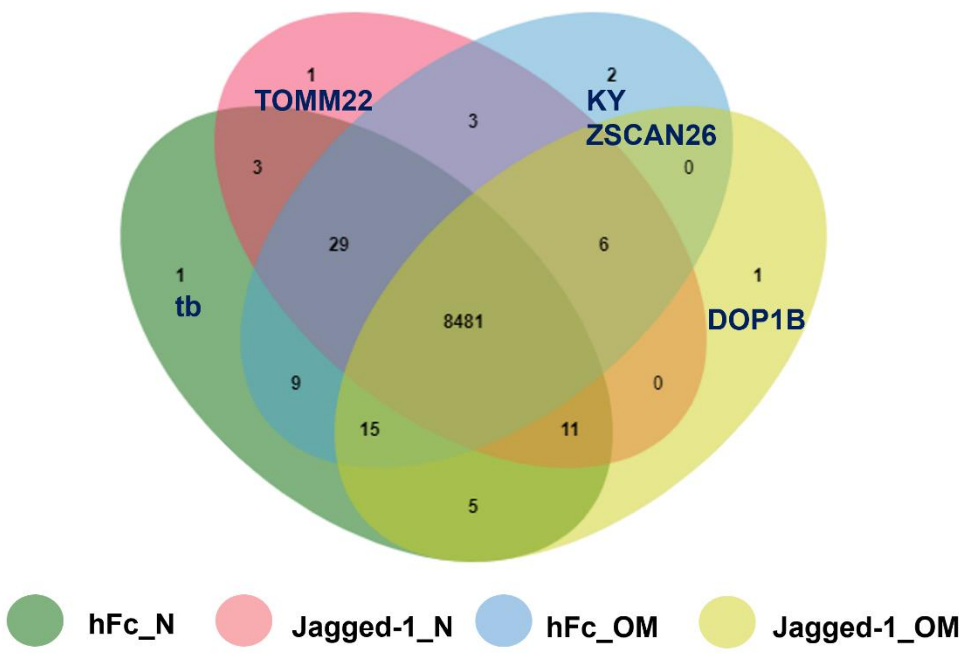

Figure S1. Venn diagram of the whole proteome classified by culture condition.

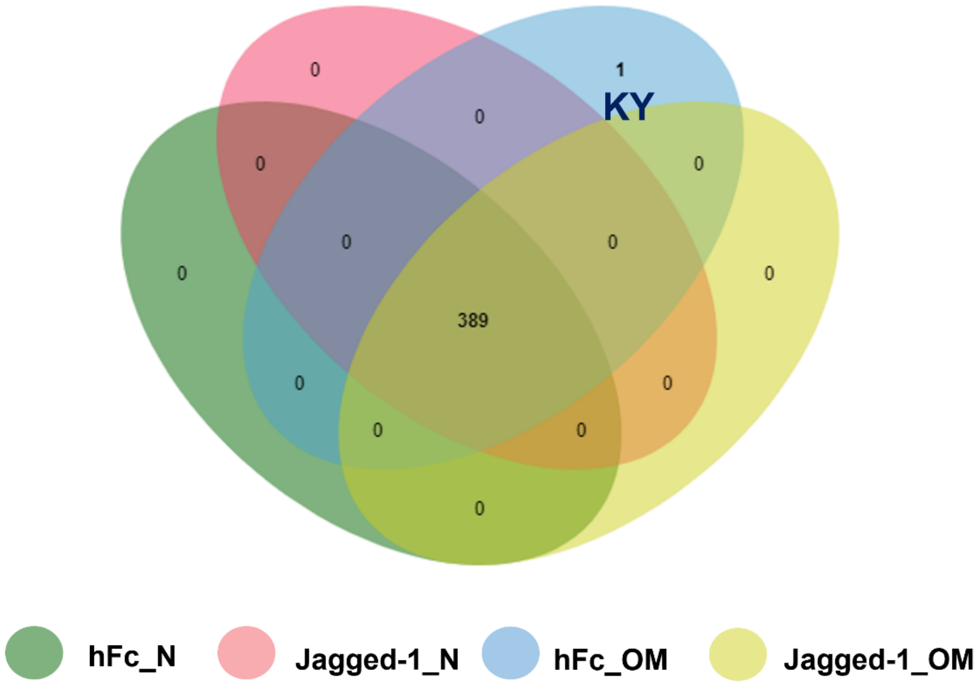

Figure S2. Venn diagram of the matrisome proteins in all cellular components separate by culture condition.
